# 

**Published:** 1894-05

**Authors:** 


### NOTICES.

### Program for Dental Section of American Medical Association.

Address, by Dr. M. H. Fletcher.

"Professional Ethics," by Dr. Van Orden.

"The Lateral Incisor," by Dr. C. L. Goddard.

"The Teeth of our School Children—What Shall be Done to Save Them?" by Dr. J. C. McCoy.

"Pyorrhœa Alveolaris, What it is and What the Dentist can do for it," by Dr. L. L. Dunbar.

"Pyorrhœa Alveolaris," by Dr. W. J. Younger.

"Remote Effects of Oral Lesions," Dr. W. B. Sherman.

"The Systemic Factor in the Treatment of Dental Caries," by Dr. G. S. Dean.

"The Present and Future Status of the Dental Practitioner," by Dr. J. D. Hodgen.

"Operations on Congenital Cleft Palate," by Dr. Robert McLain.

"Random Thoughts on our Specialty," by Dr. A. E. Baldwin.

"Pathological Notes on Neoplasms of the Maxillæ with Cases and Photographs," by Dr. Vida A. Latham, F.R.M.S., D.D.S.

"The Present Scientific Status of Hypnotism," by Dr. W. X. Sudduth.

Dr. E. S. Talbot.———

Dr. J. Taft.———

DR. M. H. FLETCHER, Cincinnati, O., Chairman.

DR. E. S. TALBOT, Chicago, Ill., Secretary.

### Chicago Dental Society.

At the annual meeting of the Chicago Dental Society, held in Kindergarten Hall, April 3, 1894, the following officers were duly elected for the ensuing year :

J. H. Woolley, President; C. E. Bentley, 1st. Vice-President; D. M. Gallie, 2d. Vice-President; A. H. Peck, Recording Secretary; H. A. Costner, Corresponding Secretary; E. D. Swain, Treasurer; J. J. Whaley, Librarian; J. M. Crouse, Director.

Yours truly,

H. A. Costner, Cor. Sec.

### The Midwinter Fair, San Francisco.

The Midwinter Fair Dental Congress, will be held at San Francisco, Cal., June next from the 11th to 15th inclusive.

All the reputable legal dentists of the nine States and Territories of the Pacific slope, together with those east of the Rockies, who wish to join with us, are expected, and urgently requested to attend this meeting, bringing with them any and everything that may be of interest to the profession.

Come prepared to give rather than to take.

Interesting papers and clinics are promised from leading men both East and West.

A meeting is anticipated that will be second only to that held at Chicago last August.

The work is being carried forward by the following officers and chairmen of committees :

President—Luther A. Teague, 10 Geary street, San Francisco.

Vice-Presidents—S. E. Knowles, 139 Post street, S. F.; J. A. W. Lundborg, 219 Geary street, S. F.

Treasurer—W. A. Knowles, 118 Grant ave., S. F.

Secretary—W. Z. King, 1001 Valencia street, S. F.

Asst. Secretary—C. W. Hibbard, 202 Stockton street, S. F.

Cor. Secretary—C. E. Post, 14 Grant ave., S. F.

Chairmen of Committees—Executive, S. H. Roberts, 120 McAllister street, S. F.; Finance, Harry P. Carlton, Crocker

Building, S. F.; Programme, Walter F. Lewis, 462½ 13th street, Oakland; Editorial, J. D. Hodgen, 1005 Sutter street, S. F.; Membership, T. N. Iglehart, 713 Sutter street, S. F.; Invitation, L. L. Dunbar, 500 Sutter street, S. F.; Reception, J. A. W. Lundborg, 219 Geary street, S. F.; Local Arrangement, F. C. Pague, 819 Market street, S. F.; Dental Club, W. J. Younger, 300 Stockton street, S. F.; Advisory, Thomas Morffew, 702 Market street, S. F.; Association Day, A. F. Merriman, jr., 1003½ Broadway, Oakland; Publication, F. Teague, 21 Powell street, S. F.; Transportation, R. H. Cool, 1115 Broadway, Oakland.

C. E. Post, Cor. Sec.

### The Dental Society of the State of New York.

The twenty-sixth annual meeting of the Dental Society of the State of New York will be held in the Lecture Room of the Young Men's Christian Association Building, Albany, N. Y., on Wednesday and Thursday, May 9 and 10, 1894.

The following interesting programme has been arranged for this occasion:

President's Annual Address, F. T. Van Woert, M.D.S., Brooklyn.

"Crown and Bridge-Work—a Protest Against Some of Its Abuses," S. H Guilford, D.D.S., Ph.D., Philadelphia.

"Can Pyorrhœa be Cured?" J. Allen Osmun, M.D.S., Newark, N. J.

"The Sixth-Year Molars," R. M. Sanger, D.D.S., East Orange, N. J.

"The Use of Peroxide of Sodium, as recommended by Dr. E. C. Kirk," W. J. Turner, M.D., D.D.S., Brooklyn.

"Green Stain in its Relation to Decalcification of Enamel," R. Ottolengui, M.D.S.

Dentists who are not members of the Society are cordially invited to be present and to participate in the discussions of the meeting; and the members are particularly requested to come prepared to assist in making the meeting one of unusual interest.

### The Texas State Dental Association.

The Annual Meeting of the Texas State Dental Association will be held in San Antonia, May 15, 1894.

A. A. Beville, President.
Chas. B. Lewis, Secretary.

### THE DENTAL REGISTER,

A MONTHLY MAGAZINE DEVOTED TO THE INTERESTS OF THE

### DENTAL PROFESSION.

*Terms Reduced to* $2.00 *Per Year. Single Copies,* 25 *Cents.*

### EDITED BY PROF. J. TAFT, D.D.S.

—AND—

### Published by SAM'L A. CROCKER & CO., Ohio Dental and Surgical Depot.

The volume commences in January and closes in December.

The REGISTER being issued monthly is always fresh, therefore Indispensable to the practitioner who desires to keep posted up with the new inventions and improvements which are daily developed. Neither expense nor effort will be spared to give value and variety to its contents Articles will be illustrated with well executed engravings whenever they will add to the interest or assist to explanation.

Its extensive circulation and frequency of issue make it one of the BEST DENTAL ADVERTISING MEDIUMS in the United States.

All subscriptions must begin with January or July.

Local or special notices, five lines or less, will be inserted for $3 each insertion; over five lines, 50 cts. per line.

All advertising bills payable in advance, or at furthest, after the issue of the first number containing the advertisement. All transient advertisements to be paid for in advance.

☞CONTRIBUTIONS SOLICITED.

Exclusively Editorial Communications should be addressed to the Editor.

The latest number of the REGISTER may be ordered with or without other good[illegible] any time, and all letters should be addressed to

